# Barriers and Enablers for Adherence to Antiretroviral Therapy Among People Living With HIV/AIDS in the Era of COVID-19: A Qualitative Study From Pakistan

**DOI:** 10.3389/fphar.2021.807446

**Published:** 2022-01-28

**Authors:** Ali Ahmed, Juman Abdulelah Dujaili, Musarat Jabeen, Malik Muhammad Umair, Lay-Hong Chuah, Furqan Khurshid Hashmi, Ahmed Awaisu, Nathorn Chaiyakunapruk

**Affiliations:** ^1^ School of Pharmacy, Monash University, Subang Jaya, Malaysia; ^2^ Department of Pharmacy, Quaid-I-Azam University, Islamabad, Pakistan; ^3^ ART Centre, Pakistan Institute of Medical Sciences (PIMS) Hospital, Islamabad, Pakistan; ^4^ National AIDS Control Programme, National Institute of Health, Islamabad, Pakistan; ^5^ University College of Pharmacy, University of the Punjab, Allama Iqbal Campus, Lahore, Pakistan; ^6^ Department of Clinical Pharmacy and Practice, College of Pharmacy, QU Health, Qatar University, Doha, Qatar; ^7^ Department of Pharmacotherapy, College of Pharmacy, University of Utah, Salt Lake City, UT, United States

**Keywords:** people living with HIV/AIDS, antiretrovirals, barriers and facilitative factors, Pakistan, challenges, interventions, UNAIDS

## Abstract

**Background:** With the increased availability of safe antiretroviral therapy (ART) in recent years, achieving optimal adherence and patient retention is becoming the biggest challenge for people living with HIV (PLWH). Care retention is influenced by several socioeconomic, socio-cultural, and government policies during the COVID-19 pandemic. Therefore, we aim to explore barriers and facilitators to adherence to ART among PLWH in Pakistan in general and COVID-19 pandemic related in particular.

**Methods:** Semi-structured interviews were conducted among 25 PLWH from December 2020 to April 2021 in the local language (Urdu) at the ART centre of Pakistan Institute of Medical Sciences, Islamabad, Pakistan. Interviews were audio-recorded in the local Urdu language, and bilingual expert (English, Urdu) transcribed verbatim, coded for themes and sub-themes, and analyzed using a phenomenological approach for thematic content analysis.

**Results:** Stigma and discrimination, fear of HIV disclosure, economic constraints, forgetfulness, religion (Ramadan, spiritual healing), adverse drug reactions, lack of social support, alternative therapies, and COVID-19-related lock-down and fear of lesser COVID-19 care due to HIV associated stigma were identified as barriers affecting the retention in HIV care. At the same time, positive social support, family responsibilities, use of reminders, the beneficial impact of ART, and initiation of telephone consultations, courier delivery, and long-term delivery of antiretrovirals during COVID-19 were identified as facilitators of HIV retention.

**Conclusion:** Improving adherence and retention is even more challenging due to COVID-19; therefore, it requires the integration of enhanced access to treatment with improved employment and social support. HIV care providers must understand these reported factors comprehensively and treat patients accordingly to ensure the continuum of HIV care. A coordinated approach including different stakeholders is required to facilitate patient retention in HIV care and consequently improve the clinical outcomes of PLWH.

## Introduction

With significant advances in antiretroviral therapy (ART), acquired immunodeficiency syndrome (AIDS) has progressed from acute to manageable chronic human immunodeficiency virus (HIV) infection, with improved immunity, viral suppression, and improved health-related quality of life (HRQoL) of people living with HIV/AIDS (PLWHA) (Kanters et al., 2017; Ahmed et al., 2021a). However, retention in HIV care has become a significant challenge, and it is seen more difficult during the COVID-19 pandemic due to lockdown and travel restrictions (Kanters et al., 2017; Jiang et al., 2020; Mhango et al., 2020). Non-adherence to ART is a common problem among HIV patients, with rates ranging from 19% in North America and Western Europe to 40% in Latin America, 28–42% in Africa, and 40% in Asia-Pacific (UNAIDS, 2021). ART-default (loss of follow-up) has been reported as an important factor leading to viral resistance, and disease progression that leads to ultimate consequences such as co-morbid conditions and mortality (Abadiga et al., 2020; Ahmed et al., 2021). HIV outbreaks are on the rise in Pakistan, which currently accounts for 0.24 million PLWHA (NACP, 2021; Ahmed et al., 2019). As of August 2021, 46,912 PLWHA registered with the National AIDS Control Programme (NACP), Pakistan, of which 26,093 are on treatment, implying that approximately 45% are not on treatment (NACP, 2021).

The research on PLWHA affected by COVID-19 is in the infancy stage; however, a recent meta-analysis reported that PLWHA are likely to have a significantly high risk of contracting COVID-19 infection [risk ratio (RR) 1.24, 95% confidence interval (CI) 1.05–1.46)] and mortality (RR 1.78, 95% CI 1.21–2.60) compared to the general population (Ssentongo et al., 2021). According to a modelling study by World Health Organization (WHO) in the Sub-Saharan African region, impediments in the supply of antiretrovirals (ARVs) and other HIV prevention services will exacerbate the situations such as; increasing the likelihood of HIV-related morbidities and new HIV outbreaks (Jewell et al., 2020). During the pandemic, all the countries have adopted measures to reduce the spread of COVID-19 infection, thus restricting a person to person physical contact by way of social distancing (Shiau et al., 2020). The measure such as Lock-down strategy are aimed at confining residents at home, thus limiting their movement. In such scenarios, provision of healthcare services to patients with chronic conditions such as HIV is affected significantly due to disruption or restriction of hospital visits, diagnosis and ultimately hamper the treatment for PLWHA (Linnemayr et al., 2020). To date limited data is available in the context of COVID-19 restrictions pertaining to PLHWA attaining ART from HIV clinics.

Adherence to ART is a dynamically complex behaviour influenced by socioeconomic as well as cultural factors (Castro, 2005). Some qualitative studies have reported barriers that include forgetfulness, fatigue, hopelessness (Jones et al., 2015), stigma and discrimination (Wasti et al., 2012), HIV non-disclosure (Arnold et al., 2014), and religious beliefs (Holtzman et al., 2015; Medved Kendrick, 2017). Facilitators of retention to treatment include family support (Yehia et al., 2015), positive relationship with healthcare providers (Nam et al., 2008), access to affordable transportation (Yehia et al., 2015), livelihood support (Wang et al., 2020), improved knowledge about the disease (Ammon et al., 2018), reminders (Nam et al., 2008), attending a support group (Wang et al., 2020), and carrying ART while away from home (Kumarasamy et al., 2005; Croome et al., 2017). Many of the reported barriers and facilitators are implicated in the Pakistani context; however, some may be unique and not reported in the literature. Therefore, in this study we are aiming to explore factors in Pakistani context.

In Pakistan, HIV testing and treatment services are provided free of cost to all PLWHA. The protection and treatment of PLWHA during the pandemic of COVID-19 is crucial (Ahmed et al., 2020b). In comparison to other low-middle income countries (LMICs), Pakistan has a strikingly different socio-cultural environment (Ahmed et al., 2021d). The HIV epidemic is more prevalent in patients who inject drugs (PWID) and reported in deported migrants (Ahmed et al., 2019; Ahmed et al., 2020b). Prevention and treatment of the growing HIV/AIDS epidemic have been difficult in Pakistan’s traditional Muslim society (Ali et al., 2021). It is difficult for PLWHA to obtain HIV testing or treatment because they are afraid of being stigmatized due to misunderstandings in traditional cultural beliefs and practices. Qualitative research is thought to be a reliable method for determining the correct cause and effect of relationships, in-depth phenomena, respondents’ thoughts, and feelings (Morgan, 2017). According to the authors’ knowledge, no qualitative study has been conducted in Pakistan to investigate barriers and ART facilitators, and there is a scarcity of knowledge about patients’ experiences with adherence to ART throughout the literature during the COVID-19 pandemic. As a result, the purpose of this study is to explore the overall barriers and enablers of adherence to ART in PLWHA in Pakistan and specific facilitators and barriers during the COVID-19 pandemic to generate purposeful identification of factors that may improve treatment outcomes.

## Methods

### Study Design and Study Setting

The research followed the COnsolidated criteria for REporting on Qualitative Research (COREQ) guidelines (attached in Supplementary File) to report this study (Tong et al., 2007).

The study was conducted in one of the largest ART centres in Pakistan situated in the Pakistan Institute of Medical Sciences (PIMS), Islamabad (Ahmed et al., 2021a; Ahmed et al., 2021d). More than 3600 PLWHA are registered in this centre, and approximately 15–20 HIV patients visit it daily for their ART refills and health-related issues. The most significant feature of this centre is that treatment is provided free of cost (Ahmed et al., 2021a). This ART centre also works in conjunction with the Department of Infectious Diseases to cater the HIV patients and other infectious diseases (Ahmed et al., 2021a; Ahmed et al., 2021e).

### Study Participants

Adults PLWHA included in the study based on 1) receiving ART from the ART Centre for at least 1 year of diagnosis; 2) being willing to be interviewed (an audio record); 3) being able to converse in Urdu (National language of Pakistan); and 4) gave written or verbal informed consent. Participants were chosen by purposive sampling, a non-probability sampling technique in which patients were selected with broad geographical provenance and have the best knowledge on study issues and approached through the case manager in the counselling room or on a telephone call. The sample size has been determined by the principle of saturation as suggested by Mason Mark (Mason, 2010). Saturation point criteria refer to “the point when new incoming data fail to produce new information to address the research question OR no new information appeared thought to be different and significant.” Patients with cognitive impairments, terminally ill, hospitalized patients, and PLWHA unwilling to participate were excluded from the study.

### Interview Guide Development and Data Collection Procedure

A semi-structured interview guide was developed based on a literature review and discussions with academics and HIV treatment experts at the ART Centre PIMS, Islamabad, to ensure that all relevant issues are included in the study guide in a culturally acceptable manner. The interview guide was validated through argumentative and cumulative methods to produce a reliable interview guide (Hashmi et al., 2017). The guide was piloted on two HIV patients to ensure that the content of the interview guide sufficiently covered all aspects of the research question, and later the guide was modified accordingly. The data from pilot interviews were not included in the final thematic content analysis (TCA). Open-ended questions with appropriate probes have led the study participants to freely express their personal experiences and factors that facilitate and impede adherence to ART. Participants were encouraged to provide as much information about the subject as possible.

Because of the high stigma associated with HIV/AIDS in Pakistan (Ahmed et al., 2021d; Hussain et al., 2021), and to maintain physical distance due to the COVID-19 pandemic, the NACP of Pakistan approved the study’s conduct, with all interviews to be conducted by a female nurse (MJ), who works as a health counsellor in the ART centre. PLWHA were familiar with her and were willing to participate in the study. She has a bachelor’s degree in nursing and has worked as an HIV counsellor in ART centre for over 10 years. She regularly sees HIV patients and counsels them on HIV disease knowledge and progression, ARV medications, and other PLWHA concerns. Before the study, she had received specialized training in qualitative interviewing methods and data collection. She gets assistance from the HIV case manager and AA for logistic support in conducting the interviews. Interviews were conducted from December 2020 to April 2021. To avoid inter-individual variability, MJ conducted all interviews. After the initial general questions, the interviewer further explored by way of probing questions. All interviews were conducted in Urdu (Pakistan’s national language) and at a time convenient for the respondents during working hours. Interviews were held using a digital audio recorder, and each interview was given a unique identifying number (pseudonym) to maintain participants’ anonymity. Because of COVID-19 restrictions, the ART Centre started sending ART courier to PLWHA, for whom it was difficult to visit the clinic and have no problem receiving medicine *via* courier. HIV case manager approached these patients and offered them a telephone interview at their ease. Three PLWHA that accepted to be the part of study their interviews were conducted on telephone call. Fieldnotes of scrupulous information were also noted, and each interview lasted from 24 to 37 min. Response saturation was reached at the 22nd interview, and three more interviews were conducted to validate the saturation of responses (Saunders et al., 2018).

### Data Analysis

Data processing was carried out manually using TCA as described by Braun and Clarke (Braun and Clarke, 2006) and commenced simultaneously during the data collection phase. All recorded interviews were transcribed verbatim by the AA. Verbatim English translation of all the transcribed interviews was undertaken by AA and further verified by FKH, MMU, and MJ. Researchers (AA, MMU, MJ, and FKH) listened to the audio recordings and read the transcripts many times to gain an in-depth understanding of the data. After extensive discussion with research supervisors (JAD, CLH, AA1), meaningful words, phrases, and sentences related to the study’s objectives were extracted manually in Microsoft^®^ Excel spreadsheets from each interview. A list of initial codes was generated (AA, MJ, FKH, MMU). Next, we grouped all the key codes based on similar features. We searched through the topics of codes by gathering all similar and repetitive codes into a sub-theme. Themes were generated by combining several sub-themes (AA, MJ, JAD, MMU, FKH, AA1). Cross-checking was undertaken to ensure data credibility and enhance trustworthiness. In case of any variation, the judgment given by the supervisors (AA1 and JAD) was considered final. Numerous direct quotes were included in the results to ensure that the results accurately reflect what respondents meant.

### Ethical Considerations, Rigour, and Trustworthiness

Ethics approval was obtained from the National AIDS Control Programme (NACP) of Pakistan and PIMS hospital, Islamabad (Approval No; 2060). The research procedures followed the Helsinki Declaration and the WHO Guide to Good Clinical Practices (World Health Organization, 2005). Patients were introduced to the purpose of the research and were consented to the audio recording of the responses. Participants were assured to keep both their identity and data confidential (Tong et al., 2007). Several methods were used to ensure the validity and rigor of the findings (Hadi and José Closs, 2016; Amin et al., 2020), including the development of a coding system, peer review of themes, sub-themes, rapport with participants, triangulation of multiple data sources (visual materials, field notes, and interview transcripts), and the provision of a detailed description that recognizes the context of data collection.

## Results

### Characteristics of Participants

The study included 25 PLWHA, with a mean age of 41 years (range 19–69), consisting of (*n* = 17) males (*n* = 10), living in a relationship (*n* = 14), having no formal education (*n* = 18), unemployed (*n* = 16), living in rural areas, and (*n* = 10) taking ART from 4 to 6 years (Table 1).

**TABLE 1:** Characteristics of study participants.

Pseudonyms	Age	Gender	Marital status	Education	Employment	Residence	Ethnicity	Time since taking ART
P1	52	Male	Married	None	No	Rural	Pashtun	9
P2	40	Male	Married	Primary	Yes	Rural	Punjabi	3
P3	24	Female	Single	Secondary	No	Urban	Punjabi	3
P4	33	Female	Married	Primary	yes	Urban	Kashmiri	2
P5	44	Female	Married	Primary	no	Rural	Pashtun	4
P6	52	Female	Widowed	None	no	Rural	Sindhi	5
P7	37	Female	Divorced	None	no	Rural	Punjabi	2
P8	64	Male	Divorced	None	no	Rural	Punjabi	6
P9	56	Male	Married	None	yes	Rural	Pashtun	5
P10	51	Male	Married	None	no	Rural	Punjabi	8
P11	69	Male	Widower	None	no	Rural	Kashmiri	7
P12	23	Male	Single	None	no	Urban	Sindhi	2
P13	19	Male	Single	None	no	Rural	Punjabi	2
P14	22	Male	Single	None	no	Rural	Pashtun	3
P15	25	Male	Single	Secondary	yes	Urban	Pashtun	2
P16	47	Male	Married	Tertiary	yes	Urban	Kashmiri	4
P17	49	Male	Divorced	Primary	yes	Rural	Punjabi	5
P18	30	Male	Single	Tertiary	yes	Urban	Kashmiri	5
P19	31	Female	Divorced	None	No	Rural	Kashmiri	3
P20	51	Female	Married	Primary	No	Rural	Sindhi	2
P21	37	Male	Single	Secondary	Yes	Urban	Kashmiri	4
P22	45	Male	Married	None	No	Urban	Kashmiri	2
P23	35	Male	Single	Primary	Yes	Rural	Punjabi	3
P24	47	Female	Married	None	Yes	Urban	Punjabi	2
P25	41	Male	Divorced	None	No	Rural	Pashtun	4

### Thematic Content Analysis

**FIGURE 1:**
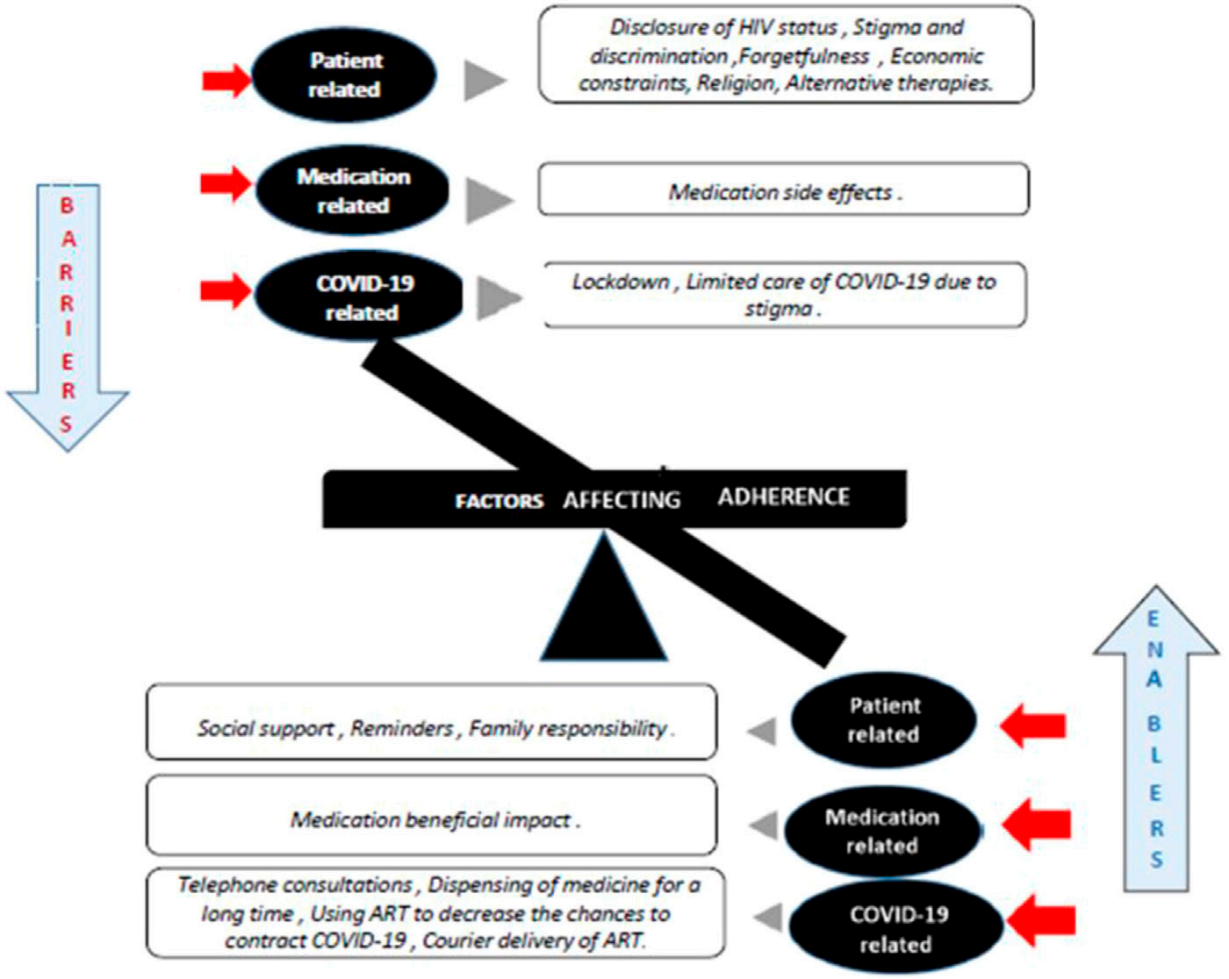
Barriers and enablers of adherence to ART identified.

The TCA yielded three major themes. 1) Patient-related factors; 2) medication-related factors and 3) COVID-19 related factors. These themes were further categorized into several subthemes, and details of these are discussed below. Tables 2, 3 presents the barriers and facilitators with selected patient quotes, respectively. Figure 1, showing the overall barriers and enablers identified in this study.

**TABLE 2:** Barriers to retention in HIV care for people living with HIV in Pakistan.

Barriers		Selected patient quotes
Themes	Sub-themes	
Patient related factors	Disclosure of HIV status	• “I am working in a marble factory; I take my medications carefully. Other office workers notice that I am constantly taking medications and point it out to me. I simply inform them that it is due to my diabetes. They’ll probably laugh in my face if I tell them it’s because of [HIV]. I may be embarrassed to get medicine and wear a mask. I’m going to die when I don’t take my medicine and, I believe, it’s not right for me to tell them I’m diseased [HIV Positive].” (P14, 22 years, male)
		• “I can’t get medicine from my own province, because I don’t want anyone in my community to know about my [HIV] status” (P9, 56 years, male)
		• “I have little trouble informing my relatives, colleagues, of my [HIV] status. When I tell them, I was encouraged to take drugs daily without suspicion and healing of my illness. However, I sometimes find it difficult to inform new colleagues because they ask many questions about how I got that [virus], and sometimes they completely avoid me in fear of getting a virus from me.” (P22, 45 years, male)
		• “I have little trouble informing my relatives, colleagues, of my [HIV] status. When I tell them, I was encouraged to take drugs daily without suspicion and healing of my illness. However, I sometimes find it difficult to inform new colleagues because they ask many questions about how I got that [virus], and sometimes they completely avoid me in fear of getting a virus from me.” (P22, 45 years, male)
		• “I think it would have been worse if I had told them because they would have told them [relatives] I should have said before.” (P5, 44 years, female)
		• “I have little trouble informing my relatives, colleagues, of my [HIV] status. When I tell them, I was encouraged to take drugs daily without suspicion and healing of my illness. However, I sometimes find it difficult to inform new colleagues because they ask many questions about how I got that [virus], and sometimes they completely avoid me in fear of getting a virus from me.” (P22, 45 years, male)
	Stigma and discrimination	• “I felt left and isolated in my family when I was sick [at the hospital]. During this time, I felt very strange because I have separate personal items, utensils and toiletries from those of the others in my family.” (P13, 19 years, male)
		• “All of my family members began to stay away from me after I received a positive test of HIV. It is so obvious: there are separated foods and utensils. They even prevent my wipes because they believe that they can be transmitted by sweat. At the next [Doctor] appointment, I talked about this scenario to doctor and the doctor guided them, no need to separate utensils.” (P15, 25 years, male)
		• “There are some [community members] who said, ‘we could get infected with the disease [HIV/AIDS].” They refused to sit besides me.” (P7, 37 years, female)
		• “Oh … It’s my fault, I think, to get HIV infected and that’s my life. I am not sure other people understand, care for and understand me about my HIV status. It is disgraceful to me for this infection, and I don’t think I should tell others.” (P24, 47 years, female)
	Forgetfulness and busy routine	• “I forget to take medicine when I am away from home or when I am depressed.” (P1, 52 years, male)
		• “I’m the only caretaker of my children [family]. I’m always very busy … I didn’t get to the hospital on time to collect medicine, I didn’t have time to eat, and I missed a few doses at different times.” (P5, 44 years, female)
		• “I usually go home late after substance abuse or alcohol intake, and no one reminds me to take ART.” (P8, 64 years, male)
	Economic constraints	• “I went to Peshawar [to find a job]. I got one time a refill from [clinic]. I worked on a farm for 2 months in the jungle. I do not speak Pashto so I could not refill the medicine. When I was travelling to Islamabad with friends, robbers stole all (my) money. Therefore, I missed my medication for 1 months.” (P1, 52 years, male)
		• “The reason for missing my medication is that … [Money]. Every month I must go to clinic for ART to cure my disease for that I need bus fare. Whose responsibility is this? No job! No money! I have no alternative but to stop treatment.” (P8, 64 years, male)
		• “Earlier, I had received ensure, a supply of supplemental nutrition for free from the NGO. But the next day, the NGO’s official told me they would no longer give it to me. I felt angry when I heard this and left the drug bottle. I didn’t take 5 days of pills because of this.” (P10, 51 years, male)
		• “I couldn’t visit [clinic], because I have no proper cloths” (P14, 22 years, male)
	Religion	• “During Ramadan, I took a break from my treatment. I was sick and went to see my doctor, and he told me not to stop treatment at any time, so I’m taking medicine now when I’m fast.” (P4, 33, female)
		• “During Ramadan, I only take the evening dose. I can’t take the dose in the morning because we only eat at night.” (P1, 52 years, male)
		• “Peer [Religious scholar] asked them to recite specific verses on the water daily and to drink that water daily for a specific period of time, [peer advised], no need to take drugs for the proper functioning of Dam Darood.” (P10, 51 years, male)
	Alternative therapies	• “Hakim told me that the virus is nothing, it’s just a weakness, he gave me some phakki (a kind of medicine) to take daily, he promised me to cure the problem fully, but after 6 months, my health started to get worse so I left the hakim phakki.” (P17, 49 years, male)
		• “Someone outside the hospital contacted me that he had created an HIV medicine that would instantly kill the virus and boost the immune system of the body, I visited clinic of this person somewhere in the rawalpidi, the doctor gave me a supplement and asked me to take it regularly. Supplement was very expensive, approximately 50,000 rupee for a month (314 USD). But after wasting so much for a year that I couldn’t recover, he also promised me that my viral load would be negative, but nothing like that happened. So after a 14-month break, I’m going to visit the PIMS again to start ART.” (P11, 69 years, male)
Medication related factors	ART Adverse effects	• “At the beginning of therapy, I had vomiting. I missed a couple of doses until I adapted to the medication.” (P1, 52 years, male)
		• “Okay, for the first time I knew, when I started using drugs, they left you, that is, sometimes it doesn’t work, I mean, even though you keep going, I know I’m in really bad mental shape the next day. They’re leaving you mentally ruined. The next day, when I take drugs, I can’t be alone! I have to be with someone, because it reminds me a lot of the first year I’ve been infected.” (P2, 40 years, male)
		• “The beginning of ART was a trying time for me. I had persistent nausea, diarrhea, and mood swings.” (P13, 19 years, male)
		• “I’ve been using ART for 6 years, in the early years I felt side effects slowly now, and now I’m used to it.” (P8, 64 years, male)
COVID-19 related factors	Impact of Lockdown	• “My transportation options to the clinic have now been narrowed down due to COVID-19. Transport is still needed because it now costs more money, or the police can hinder you. Above all, transport is not available.” (P13, 19 years, male)
		• “It’s going to have an impact on my ability, of course, because I don’t have to go anywhere. The transport had stopped. My only way to do this is to go to the clinic. I have also been worried about how I am going to be able to see you and how I’m going to be able to ask you for my medication. Some people say that the situation here is becoming increasingly grim.” (P6, 52 years, female)
	Limited care of COVID-19 due to Stigma	• “I know that one of my HIV-positive friends, who had contracted COVID-19 and had been admitted to the hospital, had been treated badly than the other person who did not have HIV.” (P18, 30 years, male)

**TABLE 3:** Facilitators to retention in HIV care for people living with HIV in Pakistan.

Facilitators		Selected patient quotes
Themes	Sub themes	
Patient related factors	Family responsibility	• “I have a boy who was born virus-free. As long as I’m alive, I want him to have better opportunities. I’m going to ruin his chance if I don’t take or give up the medications.” (P21, 37 years, male)
		• “I have two children, I am a single parent because my wife is dead, I have to take ART regularly to keep myself healthy, to work hard to make money for my children’s growth.” (P11, 69 years, male)
	Reminders	• “I’ve been busy with a lot of work; I think reminders are a great way to remember the pill I’ve got to take.” (P18, 30 years, male)
		• “To make sure that I am taking my pills on time. I have set alarm in my mobile phone.” (P16, 47 years, male)
		• “When I hear the Adhan, Allah ho Akbar, I take medicine.” (P9, 56 years, male)
	Social support	• “Initially, I failed to take my medication for a week due to a disagreement with my family; however, a doctor and a community worker advised my family not to argue, and after that, family support was critical to my adherence to therapy.” (P13, 19 years, male)
		• “My wife is HIV-negative, while I am HIV-positive. When she is angry with me because of family issues, she stops caring for me [rarely], so I miss a few doses. Her encouragement, on the other hand, is critical to my overall adherence [to treatment].” (P16, 47 years, male)
		• “Everyone in my family is extremely caring; they frequently remind me to take my medication on time.” (P12, 23 years, male)
		• “Often my children remind me to take pills when I feel tired or busy working or sleeping at a dose stage. They’re going to get me a glass of water and a bottle of prescription.” (P1, 52 years, male)
Medication related factors	Beneficial impact of ART	• “I have agreed to commit myself more to antiretrovirals because of their therapeutic benefits. I was unable to walk, but now I am healthy, and I’m usually in the office today. It is all because of the pills. I must therefore continue to take medicines to prevent disease progression.” (P18, 30 years, male)
		• “When I started the therapy, I was bedridden, having multiple diseases, high viral load, CD-4 less than 50 cells/mm^3^, than the doctor prescribed me ART, I started to recover, my CD-4 cells were raised, viral was undetected, I started to regain my health, healthier and more powerful.” (P16, 47 years, male)
COVID-19 related factors	Telephone consultations	• “You [Clinic] saved my time and money [travelling cost] and helped me improve my health through telephone call consultation.” (P6, 52 years, female)
		• “I was worried about to what additional safety measures I need to take, call [from clinic] help me to get know the disease.” (P17, 49 years, male)
	Dispensing of medicine for long time	• “My village is near Peshawar. I can’t come every month; ART centre has given me a medicine for next 6 months” (P9, 56 years, male)
	Courier delivery of ART	• “No problem. No problem. I was not [clinic] around. Two months [drug supply] they sent me a courier, and I am supposed to return in November.” (P5, 44 years, female)
		• “I was really excited to receive a call from the clinic because I ran out of ART, I lived 120 km away from the clinic, it was hard for me to visit the clinic, but thanks to the clinic staff, they sent me the medicine to my address.” (P11, 69 years, male)
		• “Staff from clinic advised me not to visit [hospital] because of increased COVID-19 cases in the country and asked about my health and other health problems and sent me the drugs *via* post office.” (P2, 40 years, male)
		• ‘‘The clinic’s nurse asked if they could send me ART by courier, but I refused because I hadn’t told anyone about my HIV status and was afraid of how people would react.’’ (P14, 22 years, male)
	Using ART to decrease the chances to contract COVID-19	• “I heard on TV that HIV people who do not take ART regularly are at risk of getting COVID-19. Therefore I am fully adherent to therapy.” (P3, 24 years, female)
		• “During the telephone consultation, Doctor advised me to adhere to ART as it would help me to fight and improve immunity against HIV and COVID-19 both. (P1, 52 years, male)”

### Theme 1: Patient-Related Factors

#### Sub-Theme 1: Disclosure of HIV Status and Social Support

Twelve participants did not even let their friends know that they were on ARVs (*N* = 3); admitted that their friends knew but were not having any idea about the disease. Patients’ treatment was of concern when others could see them taking medicines out of their homes and be suspected of living with HIV. Some patients in rural areas usually travel long distances to hospitals in other provinces to conceal their HIV status within their communities. Social stigma is another important issue that tends HIV patients to conceal it from their family members and friends. Some participants were enthusiastic and had no reservations about sharing their status with their acquaintances because they believed it would result in positive therapy outcomes in the long run (Table 2).

Participants agreed that family and friends’ social support helps to increase adherence to ART. If they forget to take medicine or visit the clinic, their family members (wife, children, parents, and relatives) remind them to remain adherent to ART. The lack of support from family members made it impossible for them to take medication because they would feel less important, uncomfortable, or sometimes forget to take medicine (Table 3).

#### Sub-Theme 2: Stigma and Discrimination

All PLWHA face discrimination in the family, society, and the anticipated stigma of HIV infection, which strongly impacts adherence to therapy. This includes segregation and division of personal belongings, and even food utensils are some of the common examples of discrimination and treatment faced by HIV patients by family members and friends. In case of discrimination by members of other communities, they did not sit close to PLWHA or move away from the PLWHA chairs and kept away from direct physical contact with PLWHA; this ultimately leads to stigma and discrimination that can promote loss to follow up. The factors that maintained the anticipated stigma among the participants were the fear of prejudice from other community members. As a result of this perception, they conceal their HIV status from members of their communities, failing to maintain regular follow-ups (Table 2).

#### Sub-Theme 3: Forgetfulness, Busy Routine, and Reminders

Participants reported that they frequently forgot their medications due to a hectic schedule or being away from home. Because single parents must work to meet their children’s basic needs, it may be difficult for them to visit the ART centre, accounting for many non-adherence cases. PLWHA who used intravenous drugs reported forgetfulness as a result of substance abuse or alcohol consumption (Table 2).

Patients often said that alarms on watches or mobile phones helped them remember to take their pills. Eleven people said they were alarmed on their mobile phones or watches. Many patients did not know how to use mobile devices or the internet, etc. Not all patients were literate, and not all of them had mobile devices. Ten of the respondents are certainly illiterate. These patients depended on unusual methods, such as the Sun’s location, the length of the shadows, the arrival and departure of students, Adhan sounds of mosques, and the bell rings of factories to take their pills (Table 3).

#### Sub-Theme 4: Economic Constraints

Participants reported that employers do not hire them due to their HIV status, which leads to unemployment, food insecurities, and distant movement to seek job opportunities. Most participants (*N* = 14) had no formal education; they often found low-wage jobs like waiters, tea boys, peons, doormen, gardeners, drivers, and sweepers at low wage rates. When they move to the countryside to find job opportunities, they face a lack of transportation, unavailability of nearby ART clinics, and language restrictions that prevent them from remaining in HIV care. Some participants reported that ART makes them drowsy, and that to combat this, they must consume expensive nutritious foods. Some also said that they are dependent on non-governmental organizations (NGOs) for the availability of food. If they found an obstruction in food, they often skip doses because they cannot tolerate medicine effects without nutritious food (Table 2).

#### Sub-Theme 5: Family Responsibility

The majority of the PLWHA were young, fertile, and had school-age children (*N* = 7). They are afraid of dying from AIDS, which would leave their children, orphans. According to the findings, PLWHA who have children are more committed to raising and educating their children, which has aided them in taking ART more seriously (Table 3).

#### Sub-Theme 6: Religion

PLWHA, a resident of a community, usually follows the religious, cultural, and traditional rituals in their everyday lives. 96.4% of the Pakistani population practices Islam, and fasting is considered as a main pillar of Islam (*N* = 11). PLWHA reported that they fast for the entire day during Ramadan and that while fasting, they do not take medication properly and usually skip the morning dose. Some participants also seek the spiritual healing to permanently end the HIV/AIDS rather than to take ART throughout the life. Two participants also reported visiting a peer (religious scholar) for Dam Darood (spiritual healing), preventing them from taking ART for 6 months (Table 2).

#### Sub-Theme 7: Alternative Therapies

Five out of twenty-five participants indicated that when doctors told them they had to take medication for a lifetime, they were concerned and tried other alternatives such as Tibb Unani, homeopathic therapy for treating HIV. Because of which they avoided taking ART for some time, but when their health got worse, they returned to the clinic for ART (Table 2).

### Theme 2: Medication-Related Factors

#### Sub-Theme 1: Side-Effects and Adverse Effects

Side effects and the adverse effects experienced during drug therapy is a normal happening. These sometimes affect the drug-taking behaviour and are often bothersome and result in failure to comply with therapy (*N* = 6). PLWHA reported that when taking ART, they usually experience unexpected or intolerable side effects, such as nightmares, psychosis, diarrhoea, and misdiagnosed psychiatric side effects, which cause them to discontinue taking medications. This is particularly common in patients who are asymptomatic and believe that taking ART worsens their health (Table 2).

#### Sub-Theme 2: Beneficial Impact of ART

The HIV status of all PLWHA in our group is confirmed after a prolonged illness. They have experienced the devastating effects of disease on their bodies, had vivid illness stories, and reported significant improvement in their health after initiating the ART. Patients reported ART improved their physical health, improved appetite, increased immunity, and prolonged life. These improvements have inspired them to take their medicines further (Table 3).

### Theme 3: COVID-19 Related Factors

#### Sub-Theme 1: Impact of Lockdown

To restrict COVID-19 spread government (Govt) of Pakistan imposed lockdown and interstate travel ban in March 2020, participants temporarily forced (*n* = 9) to become nonadherent due to finishing off their home ART stock. The most frequently reported impacts of COVID-19 restrictions were on clients’ travel to HIV clinics due to inadequate transport, police abuse, and insufficient transportation funds (Table 2).

#### Sub-Theme 2: Telephone Consultations

Participants reported that after a month of COVID-19 lockdown, the ART centre contacted them for follow-up and inquired about disease conditions and ART availability. The (*N* = 13) participants reported that telephone consultation was a great step to help them manage and get information on how to remain safe from contracting the COVID-19 (Table 3).

#### Sub-Theme 3: Dispensing of Medicine (ART) for Longer Periods

To avoid exposure to COVID-19 and to reduce the pressure of patients visiting the hospital, the ART centre prescribed the medicines for up to one to 6 months to improve the continuity of the medicine. This was done for patients who were living far away from the clinic (Table 3).

#### Sub-Theme 4: Limited Care of COVID-19 due to Stigma

The PLWHA expressed concern about being hospitalized if they contracted COVID-19. Participants reported they might be treated differently than non-HIV people due to the stigma and discrimination associated with HIV (Table 2).

#### Sub-Theme 5: Using ART to Decrease the Chances to Contract COVID-19

Most interviewers reported hearing from friends and family that PLWHA and other chronic disease patients are at increased risk of contracting COVID-19. Some also said that during consultation doctor also guided them to be adherent to ART as PLWHA are slightly at more risk of COVID-19 than ordinary people. They are regularly taking drugs to maintain their viral suppression and increase their immunity (Table 3).

#### Sub-Theme 6: Courier Delivery of ART

The clinic began courier delivery to PLWHA living in remote rural areas, and this service was only offered to those clients who agreed to receive the medicine by courier, and they appreciated this service and expressed that this service should be continued even after the pandemic. Some clients (*N* = 4) declined courier delivery because they were afraid to be exposed to people who did not know their HIV status (Table 3).

## Discussion

The exploratory nature of qualitative design has enabled us to use a phenomenology-based approach to explore the perspectives of PLWHA and generating rich data to identify the gaps otherwise overlooked by other methodological approaches in outcome research (Pietkiewicz and Smith, 2014; Queirós et al., 2017). Reducing HIV-related morbidity and mortality means ensuring that PLWHAs remain in antiretroviral therapy even during the COVID-19 pandemic, which can help prevent new cases of HIV infection and improve the quality of life of PLWHA (Mirzaei et al., 2021). We aimed to explore the barriers and facilitators that influence PLWHA adherence to antiretroviral therapy in Pakistan. Stigma and discrimination have been recognized as significant barriers, with fear of disclosure of HIV, economic constraints, forgetfulness, religion (spiritual healing), adverse drug reactions, lack of social support, and COVID-19 lockdown restrictions also affecting retention to therapy. In the meantime, positive social support, family responsibilities, reminders, the beneficial impact of ART, telephone consultation, courier delivery of ART, using ART to decrease the fear of contract COVID-19, and long-term drug delivery during COVID-19 have been recognized as facilitators of HIV retention. Studies in other resource-limited settings reported in line with these factors (Wasti et al., 2012; Bezabhe et al., 2014; Kuznetsova et al., 2016; Phuphanich et al., 2016; Chirambo et al., 2019). However, some findings such as spiritual healing, alternative treatment methods, traditional time management, job migration, and COVID-19 related factors like impact of lockdown, telephone consultations, dispensing of ARVs for longer period, courier delivery of ART and using ART to decrease the chances to contract COVID-19 have not been reported in other settings. This can be attributed to the differences in the socioeconomic status and socio-cultural norms of our study sample.

The most common barrier reported by most respondents was fear of disclosure of one’s HIV infection status and this finding is consistent with the studies conducted in other LMICs (Wasti et al., 2012; Bezabhe et al., 2014; Chirambo et al., 2019). Studies done in Sub-Saharan countries demonstrated that covert usage of ART is to delay or miss medication that ultimately leads to ART adherence failure (Croome et al., 2017). Alternatively, a prior and full disclosure of HIV status has been associated with full retention to HIV care (Chirambo et al., 2019; Dessie et al., 2019). We suggest promoting the mutually facilitated disclosure of HIV status and increasing actions such as integrating psychological health services into ART clinics would help patients navigate through acceptance of their HIV disclosure outcomes.

Stigma and discrimination appeared to be the most general barrier to ART adherence and maintenance of care. Also, Other studies in America (Earnshaw et al., 2013; Darlington and Hutson, 2017; Gunn et al., 2021; Yabes et al., 2021), Middle East (Aghaei et al., 2020; Ballouz et al., 2020; Moradzadeh and Zamanian, 2021), Asia (Ekstrand et al., 2018; Ekstrand et al., 2020; Stephens and Surjan, 2020), Sub Saharan Africa (Jones et al., 2020; Chimoyi et al., 2021; MacLean and Wetherall, 2021; Madiba et al., 2021), and Europe (Vaughan et al., 2020; Hedge et al., 2021) have found stigma to be a significant contributor to non-adherence. In Pakistan, the stigma of HIV/AIDS is enormous (Ahmed et al., 2019; Ahmed et al., 2021a; Ahmed et al., 2021d). It’s primarily due to misunderstandings about HIV risk factors and lack of knowledge of advances in treatment such that those who are treated have lowered risk of transmitting HIV (Ahmed et al., 2021f). Further, we found that PLWHA were afraid of being ostracised from their family, friends, and the public this may be attributed to poor knowledge among the public regarding the transmission of the virus. Therefore, it ultimately calls for more public education campaigns (Kumarasamy et al., 2005; Bezabhe et al., 2014; Kuznetsova et al., 2016). Discrimination towards people with HIV/AIDS is a dynamic socio-cultural phenomenon that is an outcome of viewing people with HIV/AIDS as “less than human.” (Phuphanich et al., 2016).

A multi-mechanism approach that includes the provision of information, counselling, and facilitating interaction between people who are HIV-infected and the community to reduce stigma and increase care participation is an effective HIV prevention strategy (Grossman and Stangl, 2013). A systematic review and meta-analysis by Mak et al. (2017) demonstrated that there is a lack of effective stigma reduction programmes to be implemented on a larger scale. Considering different types of health inequalities in Pakistan, addressing multi-level stigma and discrimination could improve patients’ adherence to ART treatment.

The present study explored the forgetfulness of HIV patients, which is quite common among chronic patients. Our findings on the forgetfulness of ART use are consistent with the Jones and Phuphanich et al. studies that attribute alcohol use and extra working hours to influencing PLWHA drug-taking behaviour (Bezabhe et al., 2014; Jones et al., 2015). The need to maintain private HIV status in alcohol use influences noncompliance with ART, resulting in avoiding ART when consuming alcohol (Fisher et al., 2007). Fear of the combined medicine toxicity and side-effects of alcohol also results in a default (Chirambo et al., 2019). We suggest that social habits should be included and explored as a continuous ART-care process (Mabweazara et al., 2018). To prevent drink and substance abuse-related nonadherence, Pakistan requires support groups and education intervention within ART programmes.

The lack of jobs, resulting in migration from home stations to find an appropriate position, affects adherence to therapy. Lack of proper clothing and food insecurity were among the main socioeconomic constraints that negatively affected retention of HIV treatment found in the present study that even got worse in the COVID-19 pandemic. These factors have also been reported in other studies conducted in Togo, Ethiopia and Kenya (Bezabhe et al., 2014; Yaya et al., 2014; Aibibula et al., 2017; Wang et al., 2020). Patients usually lose follow-up when they migrate either within Pakistan or overseas for work purposes (Ahmed et al., 2020b). Some studies have also documented the migration trend of Pakistani youth from rural to urban areas of Pakistan for work and the Middle East and Southeast Asian countries (Ahmed et al., 2020b; Khan and Cailhol, 2020). Bezabhe et al. (2014), reported patients usually stop taking ART medication when they cannot afford food or when NGOs no longer supply ration, especially when unemployed. Such factors are believed to exacerbate HIV-related problems, including stigma and prejudice, reduced physical activity, drug schedules, and indirect costs of care, which are not exceptional for HIV/AIDS patients. Strategies need to be implemented to improve access to jobs and food security for HIV-positive patients in Pakistan. Both government and non-governmental organizations need to combine efforts to address the multidimensional disadvantages of PLWHA.

It is important to note that religious fundamentalism is a very complex issue and often affects patients’ preferences for antiretroviral therapy in our study and this factor was also reported by studies conducted in Zimbabwe (Mutambara et al., 2021), Nigeria (Victor-Aigbodion, 2020), Ghana (Dzansi et al., 2020), Iran (Aghaei et al., 2020) and United States (Vigliotti et al., 2020; Doolittle et al., 2021). In Ramadan, Muslims observe fasting during the daytime from sunrise to sunset. Some patients usually skip medicines to fulfil their religious duties, and a similar trend is also found in other studies (Bezabhe et al., 2014; Croome et al., 2017). Members of the religious community, governments, and NGOs should work with the health authorities to help manage therapies during fasting months.

Social support and reminders are described as key enablers in this study; similar findings have been documented in other studies (Bezabhe et al., 2014; Kuznetsova et al., 2016; Mao et al., 2018). To promote adherence, HIV counsellors in our study usually advise patients to communicate their status to family members and close friends, willing to provide social assistance, pills, financial support, and emotional assistance. Dialogue and behavioural exercises are thought to improve the disclosure act effectively; some studies have documented the significance of the above (Montalto et al., 2017; Mi et al., 2020).

Electronic devices like telephones have the benefit of reminding patients when they must take their drugs while keeping the privacy of their status. Meta-analyses have shown that the short message service (SMS) on cell phones has improved compliance with ART treatment as well as HIV care retention (Kanters et al., 2017; Shah et al., 2019). Some of the traditional approaches such as the Sun’s movement during the daytime and the sounding of the prayer calls from the mosques, are usually reckoned as reminders for illiterate patients taking ART. Many HIV patients in Pakistan are illiterate and are dependent on traditional time counting methods that do not measure time by time and are influenced by many different factors. Hence, healthcare providers need to guide patients to use a quick, electronic reminder to enhance retention in care.

In our study, respondents raised difficulty reaching ART clinics during COVID-19, and a similar trend was observed in Chinese and Uganda studies (Guo et al., 2020; Linnemayr et al., 2020). Participants welcome the clinic step at COVID-19 for telephone consultations, delivery of medicine for a longer duration (up to 6 months), and appreciated the courier delivery of ART to remote patients. Similar actions have been reported by Quilantang et al. (2020) in the Philippines. Although the world has been struggling to contain the pandemic of COVID-19, millions of people are living with HIV and need constant medical supervision. There is growing concern that the response to COVID-19 may cause harm to individuals who have chronic infections.

The last decade has seen a gradual shift from hospital-based ART to primary health centres and, most recently, to the community (Kredo et al., 2013; Avong et al., 2018; Shoptaw et al., 2020). For the benefit of patients and medical professionals, community pharmacies can play a crucial role in the provision of ART services in the local community (Lelubre et al., 2018). Studies in western countries have shown that community pharmacies can play various roles in treating HIV/AIDS (Hirsch et al., 2009; Rosenquist et al., 2010; Murphy et al., 2012). Plenty of evidence suggests that trained and educated HIV pharmacists at specialized community/dispensing pharmacies and clinic-embedded pharmacist involvement in HIV care promote positive outcomes (Barnes et al., 2020). For example, Hirsch et al. (2009) found that patients who take medication therapy from trained HIV pharmacists stationed at ten community pharmacies were more likely to be classified as adherent with a medication possession ratio (MPR) of 80–120% than those who used nonspecialized community pharmacies (56.8 vs. 38.1%, *p* < 0.001) (Hirsch et al., 2009). Likewise, Cocohoba et al. (2012) compared PLWHA using HIV-focused community pharmacies with PLWHA using traditional pharmacies and found that patients using an HIV-focused pharmacy had significantly higher regimen refill adherence as measured by median MPR (90 vs. 77%, *p* < 0.0001) (Cocohoba et al., 2012). In LMICs like Pakistan, qualified clinical staff to provide optimal care, especially ART, is lacking (Ahmed et al., 2018). Suppose community pharmacists in Pakistan receive appropriate antiretroviral training. In that case, they can assist in reducing barriers to ART adherence, such as patients travelling long distances to clinics, which places a significant financial burden on patients, long waiting lines at therapy clinics at overburdened health facilities, and better pharmaceutical care. Furthermore, community pharmacists can connect with patients in highly personalised ways; they can also help improve outcomes and reduce costs associated with non-adherence, as well as provide easy access to ART in the COVID-19 pandemic (Avong et al., 2018; Ahmed et al., 2020c; Kretchy et al., 2021).

### Implications of Findings and Future Studies

There is currently very little information available on the response of people living with disabilities in the COVID-19 pandemic. Our findings highlight the key needs for the preparation of health systems to facilitate ongoing care for HIV and other chronic diseases, while transitions to normal health care are underway from the COVID-19 pandemic. We hope that the results of this qualitative study on the perceptions of ART clients about COVID-19 will provide additional valid evidence on the impact of COVID-19 on HIV care. A major advantage of this study is its contribution to a small but, hopefully, increasing literature with empirical results on how COVID-19 has a bearing on HIV care, providing first-line insights into barriers, facilitators, perception of risk, and opportunities for enhancing adherence to the PLWHA in LMICs like Pakistan. Future studies from various settings that further investigate PLWHA religious thoughts and moral stances in relation to HIV management are recommended.

### Limitations

First, we did not apply Kappa to the inter-rater reliability test, but we continued to discuss the issues through coding and thematic analysis after data collection to ensure the reliability of the data. Second, we have not collected data from bedridden patients or patients with psychiatric or other problems. Third, some interviews were done on the phone call recording; maybe there is a chance of missing some non-verbal cues, but we tried to get all the non-verbal cues on the transcript as well. Fourth, it is a single-cantered study, which may limit the generalizability of the findings, but we did our best to include PLWHA of all ethnicities. Fifth, most participants were illiterate, male, unemployed, and from rural areas, as HIV outbreaks are more concentrated in these population subsets. Lastly, COVID-19 related information is rapidly changing, and this study is cross-sectional study done during the COVID-19 pandemic, so the results should be interpreted with caution. Despite limitations, we used a purposive sampling method to identify participants with different demographic characteristics and medication-taking behaviours which best represented patients’ perspectives on the phenomenon under study.

## Conclusion

In this study, we found Stigma and discrimination, fear of disclosure of HIV infection, economic constraints, forgetfulness, religious factors, ART adverse effects, lack of social support, alternative therapies, COVID-19 related lock-downs and limited care of COVID-19 due to stigma as barriers to adherence to therapy. On the other hand, social support, family responsibilities, use of reminders, the beneficial impact of ART and initiation of telephone consultations, courier delivery, and long-term supplies of drugs during COVID-19 have improved HIV retention. To facilitate optimum adherence to ART, retention of care, and improved patient outcomes during COVID-19, interventions are needed to ensure enhanced access to health care, social acceptance of HIV, the development of social policies, and improved employment through cooperation between the various stakeholders.

## Data Availability

The original contributions presented in the study are included in the article/Supplementary Material, further inquiries can be directed to the corresponding authors (AA).
