# Supplementary material for: Barriers and Enablers for Adherence to Antiretroviral Therapy Among People Living With HIV/AIDS in the Era of COVID-19: A Qualitative Study From Pakistan
Source: Front Pharmacol. 2022 Jan 28;12:807446. doi: 10.3389/fphar.2021.807446 (PMC8832364; doi:10.3389/fphar.2021.807446)
Supplement: Supplementary file 1 [file DataSheet1.pdf]

COREQ checklist

No. Item	Guide questions/description		Location in Manuscript (Section)
**Domain 1: Research team and reflexivity**			
Personal Characteristics			
1. Inter viewer/facilitator	Which author/s conducted the interview or focus group?	MJ and assist by AA	Author contributions and Acknowledgements
2. Credentials	What were the researcher's credentials? E.g. B.A, MPhil, PhD	B.A, M.Phil. PhD	Title page
3. Occupation	What was their occupation at the time of the study?	HIV treatment experts and counsellor	Methods
4. Gender	Was the researcher male or female?	Interviewer was female	-
5. Experience and training	What experience or training did the researcher have?	Interviewer MJ has attended training sessions in qualitative studies.	-
Relationship with participants			
6. Relationship established	Was a relationship established prior to study commencement?	Yes.	-
7. Participant knowledge of the interviewer	What did the participants know about the researcher? e.g. personal goals, reasons for doing the research	Participants were encouraged to read the purpose of the study and the confidentiality statement before starting the interview. The researcher explained that she is collecting data for educational purpose.	-
8. Interviewer characteristics	What characteristics were reported about the inter viewer/facilitator? e.g. Bias, assumptions, reasons and interests in the research topic	It was explained that this research is being conducted for academic purpose. As the interviewer was working in HIV facility, she explained the participants that aim is to improve the HIV services in the Centre. No other bias was identified.	-

**Domain 2: study design**			
Theoretical framework			
9. Methodological orientation and Theory	What methodological orientation was stated to underpin the study? e.g. grounded theory, discourse analysis, ethnography, phenomenology, content analysis	Phenomenology by Thematic content analysis.	Method
Participant selection			
10. Sampling	How were participants selected? e.g. purposive, convenience, consecutive, snowball	Convenient and purposive sampling.	Method
11. Method of approach	How were participants approached? e.g. face-to-face, telephone, mail, email	22 interviews were done Face to face and 3 were done on telephone call.	Method
12. Sample size	How many participants were in the study?	25	Methods and Results
13. Non-participation	How many people refused to participate or dropped out? Reasons?	---	-
Setting			
14. Setting of data collection	Where was the data collected? e.g. home, clinic, workplace	Participants were interviewed at ART Centre PIMS and their home on telephone call.	Methods
15. Presence of non-participants	Was anyone else present besides the participants and researchers?	No.	-
16. Description of sample	What are the important characteristics of the sample? e.g. demographic data, date	Yes	Results
Data collection			
17. Interview guide	Were questions, prompts, guides provided by the authors? Was it pilot tested?	Yes.	Methods
18. Repeat interviews	Were repeat interviews carried out? If yes, how many?	There were no formal repeat interviews. However, after listening the recordings again and again, one participant was contacted to clarify his/her response.	-
19. Audio/visual recording	Did the research use audio or visual recording to collect the data?	Interviews were audio recorded.	Methods

20. Field notes	Were field notes made during and/or after the interview or focus group?	Yes.	Methods
21. Duration	What was the duration of the inter views or focus group?	The duration of interviews was in 24-37min range	Methods
22. Data saturation	Was data saturation discussed?	Yes.	Methods
23. Transcripts returned	Were transcripts returned to participants for comment and/or correction?	No. Participants were offered to read the transcripts but none of them were willing.	-
**Domain 3: analysis and findings**			
Data analysis			
24. Number of data coders	How many data coders coded the data?	All the authors (AA, MJ, JAD, MMU, FKH, AA1) except LHC, NC participated in data coding	Authors' contributions and Methods
25. Description of the coding tree	Did authors provide a description of the coding tree?	Yes.	Methods
26. Derivation of themes	Were themes identified in advance or derived from the data?	Derived from the data.	Methods
27. Software	What software, if applicable, was used to manage the data?	None.	-
28. Participant checking	Did participants provide feedback on the findings?	No one was interested to provide feedback	-
Reporting			
29. Quotations presented	Were participant quotations presented to illustrate the themes/findings? Was each quotation identified? e.g. participant number	Yes.	Table 2-3
30. Data and findings consistent	Was there consistency between the data presented and the findings?	Yes.	Results and discussion
31. Clarity of major themes	Were major themes clearly presented in the findings?	Yes.	Discussion
32. Clarity of minor themes	Is there a description of diverse cases or discussion of minor themes?	Yes.	Discussion

Interview guide

Title: Barriers and enablers for adherence to antiretroviral therapy among people living with HIV/AIDS in the era of COVID-19: a qualitative experience from Pakistan

Name of interviewer..

Date of interview...

Interview code...

Socio-demographic information

a) **Gender** ☐ Male ☐ Female

b) **Age ………………………**

c) **Education** ☐ none ☐ primary ☐ secondary

☐ University or college

d) **Employment:** ☐ Unemployed ☐ self-employed
☐ Employed by government ☐ Employed in private

e) **Residence** ☐ rural ☐ urban

h) Duration on Therapy………………………………………………

i) Marital status…………………………………………………………

1) How do you evaluate your experience in taking antiretroviral therapy (ART)?

[leave respondent to explain then after you can probe for the following].

A. individual Factors

I. Could you please share with me what you know about your ART treatment? [Allow respondent to explain then probe for prolong life, lifelong treatment, improve health status, side effects, risk of missing doses, benefits of ART, the importance of adherence to treatment and consequences of ART]

II. Could you please tell me what you do when you forget to take your ARV drugs? [take double dose next day, take immediate dose when you got to know]

III. Do you think this disease is punishment for your deeds? [corruption, lied to someone, didn't respect others]

IV. Do you think religious belief will help you to get rid of this disease?

V. Please share why people stop using ART? [Probe on tiredness with medication, feeling better, use of alternative medicines]

B. Socio-economic & community

I. What kind of support do you receive concerning your ARV medication? [Probe for type of support from family, community, friends and groups/ program]

II. Explain in detail how you manage your visits to the clinic for your appointments. [probe for transport, work situation]

III. Who have you shared with your HIV status? [Probe if this could have any effect on the respondent's adherence to medication like rending for taking medicine]

IV. In which situations have you not been able to take your ARV drugs? [Probe on fearing from being disclosed and stigmatized or lack of food/hunger]

C. Financial Factors

I. Can you explain how do you feel about the services that you are receiving from this clinic? [Allow respondent to explain then if necessary, probe on transportation costs, prescription charges, food costs, hospital diagnostic costs, service fee.

D. Health system

I. How is the confidentiality, privacy and interaction with staff? [Polite, respectful, rude]

II. What kind of support do you get from the staff? [Probe on staff behaviour on asking question, guide your medication side effects?]

E. Drug Regime Factors

I. Can you share why you started therapy and your experience concerning the ARV drugs you are taking? [If appropriate probe on number of pills, colour of pills, ARV stock-outs, size and smell of pills, dietary restriction and side effects, CD 4 count, Viral load]

II. How do you feel since you have started taking ARV? [Probe on happiness, sadness, depressed; loneliness, stress]

F. COVID-19 related factors

I. How COVID-19 affected your medication retention? [Lockdowns, corona fear, people behaviour, ART centre measures]

II. How ART centre and your family supporting you during COVID-19 [Stigma, travelling, food, ART delivery, telephone consultations, knowledge about ART].

Do you have any question for me?

Thank you very much for your time and good collaboration
